# Comprehensive search for assessment indicators that influence the level of handwriting difficulties among children in educational settings

**DOI:** 10.1038/s41598-025-03634-z

**Published:** 2025-07-02

**Authors:** Shuhei Takahata, Hiromichi Hagihara, Hiroyuki Ishihara, Daiki Enomoto, Naoto Ienaga, Haruka Noda, Shoichi Ishida, Kei Terayama

**Affiliations:** 1 Department of Occupational Therapy Faculty of Health Science, Aino University, 4-5-4, Higasiota, Ibaraki, Osaka 567-0012 Japan; 2 Graduate School of Medical Life Science, Yokohama City University, 1-7-29, Suehiro-cho, Tsurumi-ku, Yokohama, Kanagawa 230-0045 Japan; 3 Graduate School of Human Sciences, University of Osaka, 1-2, Yamadaoka, Suita, Osaka 565-0871 Japan; 4 Independent Researcher, Kanagawa, Japan; 5 LITALICO Inc., 2-1-1, Kamimeguro, Meguro-ku, Tokyo 153-0051 Japan; 6 Institute of Systems and Information Engineering, University of Tsukuba, 1-1-1, Tennoudai, Tsukuba, Ibaraki 305-8573 Japan

**Keywords:** Handwriting difficulties, Assessment, Elementary school children, Teachers’ perception, Psychiatric disorders, Human behaviour, Development of the nervous system, Somatosensory system

## Abstract

Handwriting difficulties are a major public health problem among school-aged children worldwide, as they seriously interfere with their academic performance and well-being. Teachers’ assessments of handwriting difficulties determine whether a child should be subsequently referred for medical or professional assistance. However, very few studies focused on teachers’ perception of handwriting difficulties and the relationship between such perception and direct assessments of children’s relevant abilities. As a result, the direction of support in educational settings remains vague and less focused. In this study, we investigate the relationship between teachers’ perceptions of children’s handwriting difficulties and children’s abilities relevant to their handwriting skills in three main areas: (1) handwriting process, (2) legibility of written letters, and (3) the cognitive and motor functions. The findings suggest that the presence of handwriting difficulties is related to clumsiness of foundational motor skills and the severity of handwriting difficulties is related to lack of carefulness. In addition, somatosensory and visuomotor integration were found to important indicators that characterize handwriting difficulties. These results can provide guidance to teachers and professionals who support children with handwriting difficulties, with individualized and optimal strategies.

**Supplementary Information:**

The online version contains supplementary material available at 10.1038/s41598-025-03634-z.

## Introduction

Despite the recent trend toward digitalization in education, handwriting remains important for taking notes and writing letters to friends and teachers^1–3^. Difficulties in handwriting can affect other academic skills, such as reading comprehension, memory, motivation, participation in schoolwork, academic performance, happiness, and building peer relationships^3–6^. Therefore, identifying children with handwriting difficulties at an early stage and offering them timely support is crucial.

Handwriting or writing difficulties have been identified in both medical and educational settings. In the medical field, writing difficulties are diagnosed as “impairment in written expression” under the category of Specific Learning Disorders (SLD) in the Diagnostic and Statistical Manual of Mental Disorders, Fifth Edition (DSM-5)^7^. Impairment in written expression indicates inaccurate or poor spelling, inaccurate grammar and punctuation, and difficulty organizing written expression. The prevalence of SLD, which includes dyslexia, dyscalculia, and impairment in written expression, has been reported to be 5% to 15%^7^. In educational settings, no clear terminology or definitions have described handwriting difficulties. The percentage of children teachers perceive to have handwriting difficulties is approximately 23%, which is much higher than the prevalence indicated by medical diagnostic criteria^8^. Therefore, teachers need to judge whether medical follow-up is required for children with handwriting difficulties based on the degree of difficulty and their learning situation, providing guidance accordingly. For the early detection and support of children with handwriting difficulties, investigating handwriting difficulties as perceived by school teachers is essential to establish points for detection and directions for guidance^9^.

The current status of research and implementation for the early detection of and support of handwriting disabilities can be summarized as follows. Common symptoms, such as messy handwriting, misspaced letters, and spelling problems, are usually recognized by parents or teachers^9^. The children exhibiting such symptoms are then assessed by occupational therapists, speech-language pathologists, psychologists, or other medical professionals and primarily diagnosed by a pediatrician or child psychiatrist specializing in neurodevelopmental disorders^10^. It is not only time consuming but also requires assessment by multiple professionals, hindering early detection and support. In some countries, medical professionals do not exist in educational settings. For example, in Japan, parents or teachers who notice handwriting difficulties in children need to visit medical institutions by themselves; occupational therapists and speech-language pathologists can only provide support after a diagnosis and prescription are provided by a medical doctor, delaying assistance^11^. In recent years, the combination of digital tablets and machine learning algorithms has attracted attention as a simple, convenient way to assess children with handwriting difficulties^12^. Machine learning algorithms have been developed to automatically extract features related to the legibility of written letters obtained during the handwriting process that could not be obtained with paper and pencil. These features are used to identify dysgraphia. Recent review articles have shown that machine learning-based classification can identify dysgraphia with a high accuracy of 92%–99%^13–16^. Commercial products have been developed based on these research findings, making it possible to conduct screening tests at home and in educational settings to confirm dysgraphia.

Despite the rapid progress in early detection and support through the use of digital tablets and machine learning algorithms to assess dysgraphia, two issues remain: First, because previous studies focused on “dysgraphia” based on medical test results, “handwriting difficulties” in educational settings have been overlooked, resulting in the failure to identify mild symptoms for which some support is necessary. In the majority of cases, handwriting difficulties among children in the early grades of elementary school disappear as they move to higher grades. However, handwriting difficulties sometimes persist for a certain proportion of those children, causing significant difficulties in their lives and learning^17^. No previous research has focused on teachers’ perceptions of both handwriting difficulties and children’s handwriting-related skills in detail. Understanding how teachers perceive handwriting difficulties and what underlying abilities they associate with them is essential for establishing effective early detection and support systems within educational settings. Second, examinations of cognitive and motor functions, including those that provide a broader developmental basis that is assumed to be related to handwriting difficulties, are scarce. Previous studies have demonstrated the usefulness of machine learning algorithms in predicting the presence of handwriting difficulties; however, the features used in such studies are only indicators related to the legibility of written letters and indicators in the handwriting process. Children’s motor and perception skills also relate to handwriting performance, such as postural balance functions that support hand manipulation^18^, somatosensory functions that enable fine manipulation of the fingers^19,20^, and visual functions that lead to upper limb manipulation^3,21^. However, no studies have used machine learning algorithms to predict handwriting difficulties, using these indicators, and thus the extent to which handwriting difficulties can be predicted from handwriting-related functions remains unclear. Therefore, it is valuable to investigate whether handwriting difficulties can be predicted based on these comprehensive indicators, as this could provide insights into the underlying mechanisms of handwriting performance.

This study aims to determine the relationship between children’s handwriting difficulties based on classroom teachers’ perceptions and comprehensive indicators that are considered to constitute handwriting difficulties. To achieve this goal, we conducted semi-structured interviews with classroom teachers and rated the degree of each child’s handwriting difficulties. We also broadly assessed children’s handwriting-related skills and extracted 22 indicators targeted at three organization levels: handwriting process, legibility of written letters, and handwriting-related functions, all of which are components of handwriting. We then analyzed the relationship between classroom teachers’ assessment of handwriting difficulties (3 levels) and the characteristics of the target children’s handwriting (22 indicators) using two algorithms: principal component analysis (PCA) and LightGBM^22^. The former was used to analyze the 22 indicators associated with handwriting difficulties, enabling a comparison of their relationships with the severity of handwriting difficulties as perceived by teachers. This analysis provides valuable insights for the early detection and development of targeted instructional strategies. Additionally, the latter allows us to visualize and detect the key indicators related to handwriting difficulties. This approach supports a prioritized assessment, focusing on indicators that are most likely to contribute to detecting the level of handwriting difficulties, thereby facilitating more effective interventions.

## Methods

### Participants

A total of 145 second-grade Japanese elementary school students (77 boys and 68 girls; 142 right-handed) enrolled in regular classes participated in this study. The participants’ mean age and standard deviation could not be calculated as the detailed profile information, including data of birth, was not collected due to the request of the school. Second graders were targeted because in children with typical development, handwriting quality develops rapidly in the first grade (ages 6–7), reaches a plateau by the second grade (ages 7–8), and becomes automatic, with an emphasis on cognitive processing, such as written expression, in the third grade (ages 8–9)^3,17^. Thus, second graders are considered to have relatively high individual differences in handwriting ability and are not significantly influenced by the developmental process. All second-year elementary school students enrolled in regular classes and for whom the consent of the students and their parents has been obtained are participants in this study. Thus, some of them may have intellectual developmental disorders or neurodevelopmental disorders.

### Procedure

At the partner elementary school, a preliminary explanation was given to the students, as well as the three second-grade homeroom teachers, the principal, and the vice principal, to promote sufficient understanding of the project. Data collection for the children took place during morning class hours. The tasks that were permitted to be administered in a group setting according to the evaluation manual were conducted simultaneously in each classroom, while tasks requiring individual assessment were conducted in a separate room, where the target children took turns completing the tasks. This study was conducted in accordance with the tenets of the Declaration of Helsinki, and written informed consent was obtained from parents and oral informed consent was obtained from the children. This study was approved by the Research Ethics Committee of Hakuho College (Approval No. 19024).

### Evaluation of handwriting difficulties

Handwriting difficulties were evaluated by five teachers: the classroom teacher, two teachers in charge of the same grade, the principal, and the vice principal. Children who had no handwriting difficulties were rated as “None,” children who had some handwriting difficulties were rated as “Mild,” and children who had considerable handwriting difficulties were rated as “Severe.” Each child’s rating was independently evaluated by the five teachers, after which they held a discussion to determine a final, consensus-based evaluation. This final rating was not an average of the individual scores. This evaluation method was adopted to ensure an objective assessment while also respecting the perception of the homeroom teacher, who was most involved with the target child.

### Indicators and methods of assessment

We extracted 22 indicators that were closely related to handwriting ability and handwriting-related functions and classified them into three handwriting organization levels based on indicators used in previous studies^3,18–21^: assessment of the handwriting process (operational level), assessment of the legibility of written letters (representational level), and assessment of handwriting-related functions (functional level). These indicators were selected from indicator candidates based on the following three criteria: whether they were used in multiple previous studies, whether the necessary equipment for data collection was available, and whether the evaluation could be conducted within a short time frame due to the limited time available for interaction with elementary school children. All indicators and their assessment methods are listed in Table 1.

**Table 1:** Handwriting organization levels, indicators, and the method used to assess the indicators.

Handwriting organization level	Indicator	Method of assessment
Assessment of the handwriting process (operation level)	Pen tilt_x (mean)	The mean and SD of pen tilt in the horizontal direction with the right direction representing the positive value^13^
	Pen tilt_x (SD)	
	Pen tilt_y (mean)	The mean and SD of the pen tilt in the vertical direction with the upward direction representing the positive value^13^
	Pen tilt_y (SD)	
	Handwriting pressure (mean)	The mean and SD of the handwriting pressure during the period when the digital pen is in contact with the tablet^13^
	Handwriting pressure (SD)	
	Handwriting speed	The total time spent to write the task sentence (10 letters)^13^
	Ratio of movement phase	The proportion of time spent in the movement phase—defined as the duration when the pen is in contact with the tablet and moving^13^ relative to the total time
Assessment of legibility of the written letter (representation level)	Letter ratio (mean)	The ratio of the lengths of the vertical and horizontal lines of the rectangle that surrounds the letter outline with straight lines and the mean of the absolute value of 1 (ratio of the height and width of the letter) over the 10 letters^24^
	Letter size (SD)	The area of a rectangle with the letter outline surrounded by a straight line, and the SD of the 10 letters^24^
	Letter spacing (SD)	The shortest distance between letters and the SD between the10 letters (9 sections)^24^
	Letter alignment (total)	The distance between the center point of the first letter and the that of the second and subsequent letters, and the total of the 10 letters^24^
Assessment of handwriting-related functions (functional level)	One arm and one leg balance (total time)	A subtest of the postural balance function in the JPAN^25,26^, assessing the time taken to hold one hand and one contralateral leg extended horizontally from the crawling posture^18^
	Thumb confrontation test	A sub-item in the clinical observation test^27^. Tapping is performed by the thumb and other fingers in sequence, and its accuracy and smoothness is assessed on a 3-point scale according to the evaluation criteria^18,19^
	DEM (total time)	Only TestC in the DEM^28^, which has the highest eye movement load, was selected. Total time (eye movement fluency) and number of mistakes (eye movement accuracy)^21^ are calculated
	DEM (mistakes)	
	WAVES_Line tracing (achievements)	Two items of the (WAVES^29^, line and shape tracing(subtests related to eye-hand coordination)—are administered. In the line tracing task, participants manipulate a pencil so that it does not extend beyond the presented line. In the shape tracing task, participants are asked to manipulate the pencil so that it does not extend beyond the small shapes (triangles, squares, and circles) presented to them. For each test, the number of achievements (how much progress was made), number of passes (how accurately the pencil was moved on the line), and ratio (number of passes/number of achievements)^21^ are calculated. See Fig S1 in Supplementary Information for details
	WAVES_Line tracing (passes)	
	WAVES_Line tracing (ratio)	
	WAVES_Shape tracing (achievements)	
	WAVES_Shape tracing (passes)	
	WAVES_Shape tracing (ratio)	

DEM: Developmental Eye Movement; SD: standard deviation; WAVES: Wide-range Assessment of Vision-related Essential Skills.

### Handwriting process (operational level)

**Fig. 1:**
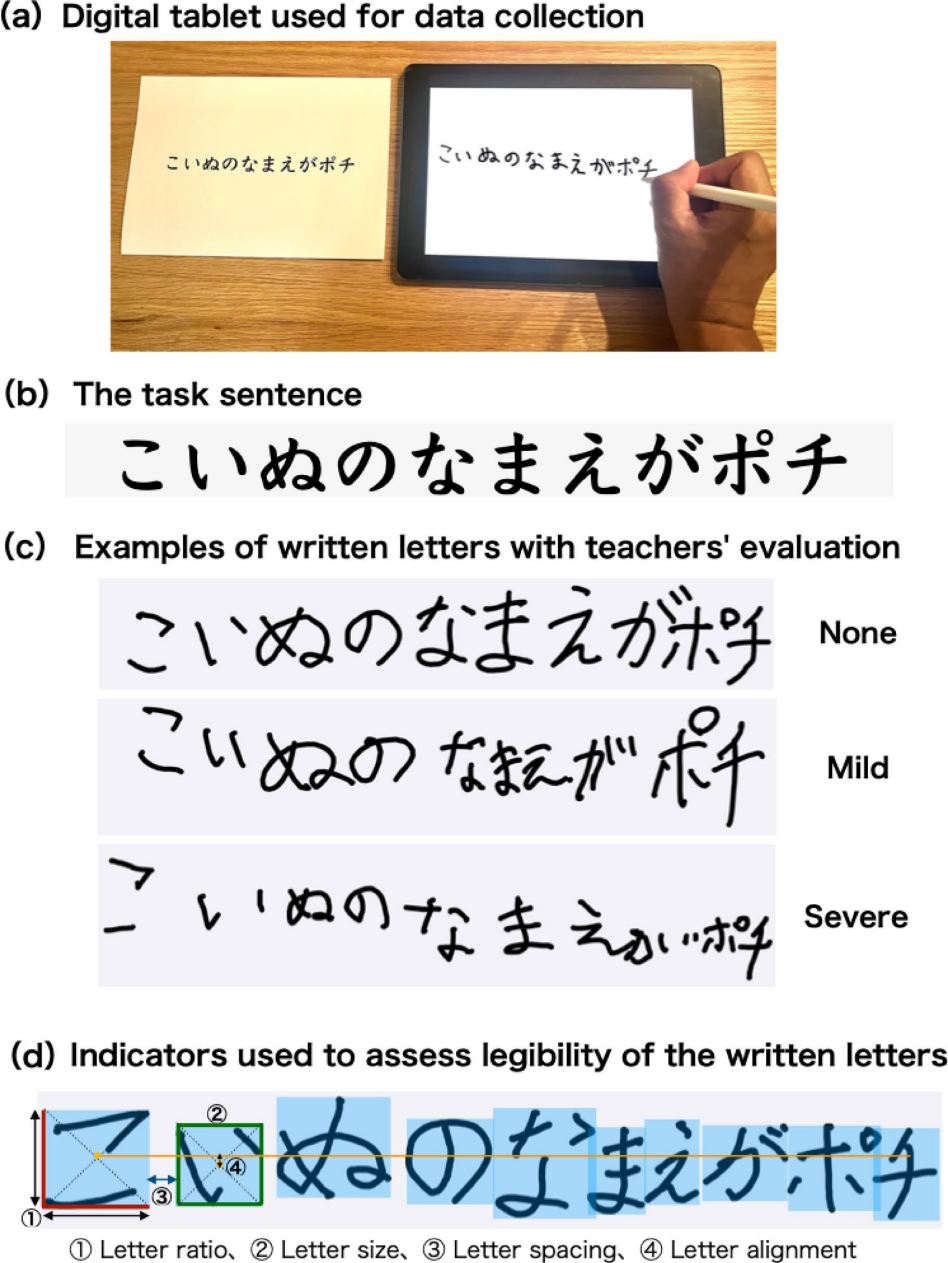
The task sentence and its evaluation. (**a**) Digital tablet used for data collection. We used an application using an iPad and Apple pencil developed by our research team. (**b**) The task sentence (in Japanese). This single sentence consists of 10 letters, including the typical morphological features in Japanese letters. (**c**) Three samples of actual letters written by children. Children who wrote these samples were rated as None, Mild, and Severe by the teachers based on those children’s handwriting performance in everyday school lives. (**d**) Indicators used to evaluate the legibility of written letters. (1) Letter ratio: ratio of length to width, (2) Letter size: area around the perimeter of the letter, (3) Letter spacing: shortest distance between letters, (4) Letter alignment: difference between the center point of each letter based on the center point of the first letter.

We used a digital tablet to assess the handwriting process during the single-sentence transcription task, as shown in Fig. 1a. The handwriting was recorded using an iPad (eighth-generation) and Apple pencil (first-generation) and analyzed using the application tool we developed^23^. The task sentence, meaning “The puppy’s name is Pochi,” consists of 10 Japanese letters, including almost all typical morphological features. Figure 1b depicts a sentence given as a reference, which each child must copy onto the tablet. Figure 1c depicts examples written by children and teachers’ evaluations. The digital tablet and the application allowed us to obtain the position of the pen tip coordinates and pressure (60 Hz), Pen tilt_x-axis (mean and standard deviation [SD]), and Pen tilt_y-axis (mean and SD), with which we calculated handwriting pressure (mean and SD), handwriting speed, and ratio of movement phases. Handwriting pressure values are normalized and its upper limit is 4.166. Even if the pressure is high, the score is 4.166 and the upper limit is reflected.

### Legibility of written letters (representational level)

We assessed the legibility of letters that appeared during the copying task. Referring to Feder and Majnemer^24^, the legibility of written letters was assessed in terms of letter ratio, size, spacing, and alignment (Fig. 1d).

### Handwriting-related functions (functional level)

Many foundational gross and fine motor skills are relevant to handwriting abilities. In this study, we focused on postural balance function, the perception of somatosensory information, and vision-related functions, which are considered as important foundational skills for handwriting^3,18–21^. The tasks corresponding to each of these, which are commonly used in Japanese clinical practice, were also used in this study. Postural balance function was examined using the one arm and one leg balance, a part of the Japanese Playful Assessment for Neuropsychological Abilities (JPAN)^25,26^. Somatosensory information was examined using the thumb confrontation test, a part of the clinical observation test^27^. Eye movements in the vision-related function were examined using Test C, a part of the Developmental Eye Movement (DEM) test^28^. Visuomotor integration was assessed using line and shape tracing, parts of the Wide-range Assessment of Vision-related Essential Skills (WAVES)^29^. See Supplementary Information for details on these assessments.

### Data analysis

We performed data analysis using PCA^30^ and LightGBM^22^-based model construction to investigate the relationship between classroom teachers’ ratings of handwriting difficulties (three levels) and the characteristics of the target children’s handwriting (22 indicators). Each indicator was standardized before being put in the models. PCA is a technique for dimensionality reduction in high-dimensional data. The vector with the largest variance in the data is set as the first principal component (PC1), and the next vector with the largest variance, orthogonal to PC1, is set as the second principal component (PC2). In addition, a one-way analysis of variance (ANOVA) was performed to determine whether there were significant differences in the degree of handwriting difficulty based on the extracted PC1 and PC2.

LightGBM is a decision-tree-based ensemble model for classification and regression prediction that performs well in terms of prediction accuracy, model stability, and computational efficiency^22^. In addition, the important features for prediction can be quantified using a method called “feature importance.” In this study, we used LightGBM to construct two models that predicted the two classification types of handwriting difficulties evaluated by teachers from the 22 indicators. These classification types were used for the predictions based on the presence of handwriting difficulties: None and the other groups (Mild and Severe) and for classification by the degree of handwriting difficulties: Severe and the other groups (None and Mild). Important indicators for the predictions were analyzed using feature importance from the two classification models.

## Results

### PCA-based analysis of assessment indicators that affect handwriting difficulties

**Fig. 2:**
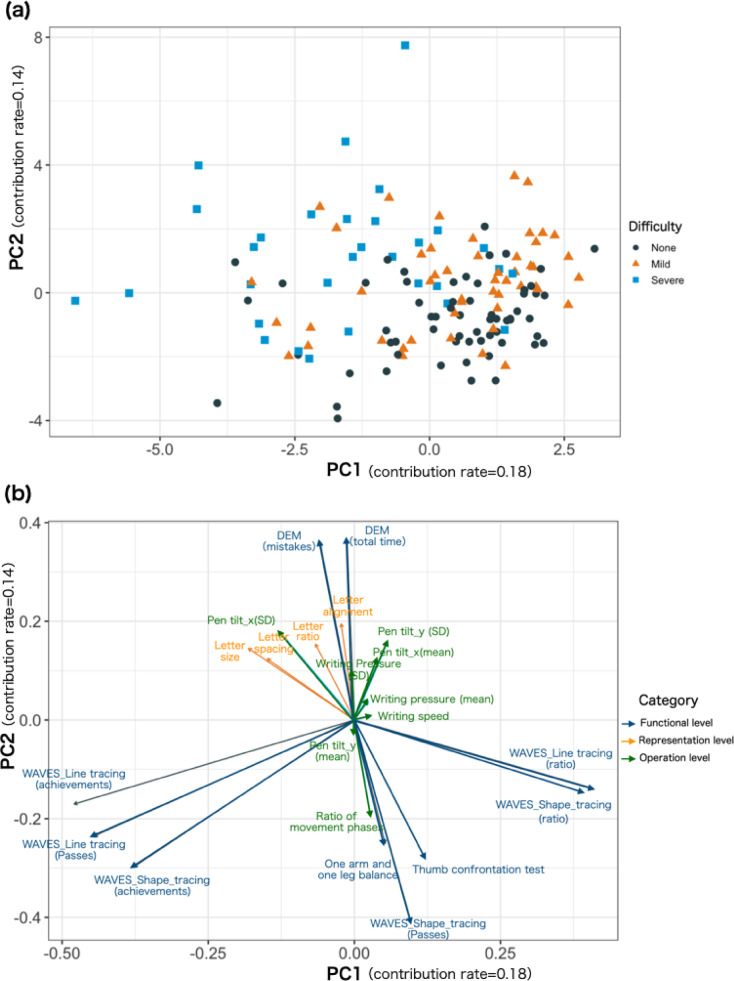
(**a**) Scatter plot of children’s data projected onto the space defined by the first principal component (PC1) and the second principal component (PC2). The black circles, orange triangles, and blue squares represent children classified as None, Mild, and Severe, respectively. (**b**) The contribution of each assessment indicator to the PC1 and PC2. The blue, orange, and green vectors indicate handwriting -related functions (functional level), legibility of the written letters (representation level), and handwriting process (operation level), respectively.

We conducted an analysis using PCA with the 22 indicators as variables. Figure 2a depicts a scatter plot of children’s data projected in the space defined by the first principal component (PC1) and second principal component (PC2). Children who were evaluated as None were mainly distributed in the positive region of PC1 and negative region of PC2, whereas those who were evaluated as Severe tended to be distributed in the negative region of PC1 and positive region of PC2. Figure 2b depicts the contributions of each indicator to PC1 and PC2.

PC1 can be interpreted as a component of “carefulness of handwriting.” The indicators with vectors extending toward the axial direction on PC1 are related to WAVES_Line tracing and WAVES_Shape tracing, which require careful handwriting between lines. Specifically, WAVES_Line tracing (ratio) and WAVES_Shape tracing (ratio), with vectors extending toward the positive direction, can receive high scores by performing the task slowly and carefully. However, WAVES_Line tracing (achievements and passes) and WAVES_Shape tracing (achievements), whose vectors extend toward the negative direction, are indicators in which speed results in higher scores, even if the tracing is less accurate. In other words, it is assumed that the positive direction of PC1 indicates the carefulness of the handwriting, whereas the negative direction indicates cluttered handwriting. See Fig. S1 in Supplementary Information for details on representative cases in WAVES_Line tracing and WAVES_Shape tracing.

PC2 can be interpreted as a component of “clumsiness of foundational motor skills”. The indicators with vectors extending toward the axial direction of PC2 are eye movement, somatosensory perception, accuracy of visuomotor integration, and postural balance function. Specifically, the DEM (total time) and DEM (mistakes), whose vectors extend toward the positive direction, indicate that the higher the value, the more difficulty the participant has with eye movements. However, the smaller values for the thumb confrontation test, WAVES_Shape tracing (passes), and one arm and one leg balance, whose vectors extend toward the negative direction, indicate that the children are better at exercising. In other words, the positive direction of PC2 indicates motor inaccuracy and clumsiness, whereas the negative direction indicates motor accuracy and dexterity.

**Fig. 3:**
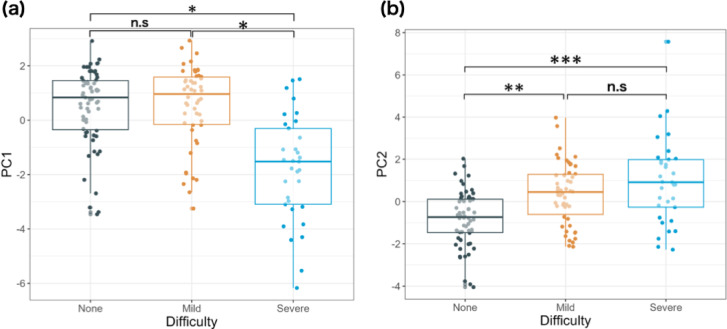
Relationship between handwriting difficulties and (**a**) PC1 (carefulness of handwriting) and (**b**) PC2 (clumsiness of foundational motor skills). The horizontal line in each box indicates the median, and the upper and lower ends of the box indicate the interquartile range. Significant differences were observed between Severe and the others for PC1 and between None and the others for PC2.

Furthermore, the relationship between the principal components and teachers’ ratings of handwriting difficulties was analyzed. Figure 3 depicts the distributions of PC1 and PC2 values for the None, Mild, and Severe groups in boxplots. The results of a one-way analysis of variance (ANOVA) revealed that PC1 and PC2 values were significantly varied across the handwriting difficulties perceived by teachers (PC1: *F*(2,143) = 27.11, *p* < 0.001, η^2^ = 0.27; PC2: *F*(2,143) = 16.94, *p* < 0.001, η^2^ = 0.19). Multiple comparisons with Bonferroni adjustment were performed to examine pairwise differences in PC1 and PC2 values among the None, Mild, and Severe groups (Fig. 3).

With regard to PC1 (carefulness of handwriting), the PC1 scores of children evaluated as Severe were predominantly lower than those of the other two groups (None: *t*(143) = 6.73, *p* < 0.001, *d* = 1.45; Mild: *t*(143) = 6.56, *p* < 0.001, *d* = 1.47), while no significant difference was observed between None and Mild (*t*(143) = 0.13, *p* = 1.0, *d* = 0.03), indicating that it may not be a factor that separates the two groups. In other words, the difference between Mild and Severe in terms of the degree of handwriting difficulties is the carefulness of handwriting, suggesting that lack of carefulness in handwriting may affect the severity of handwriting difficulties.

With regard to PC2 (clumsiness of foundational motor skills), the PC2 scores of children evaluated as None were significantly lower than those in the other two groups (Mild: *t*(143) = 4.06, *p* < 0.001, *d* = 0.77; Severe: *t*(143) = 5.43, *p* < 0.001, *d* = 1.17), while no significant difference was observed between Mild and Severe (*t*(143) = 1.77, *p* = 0.23, *d* = 0.40), indicating that it may not be a factor that separates the two groups. In other words, these results suggest that clumsiness of foundational motor skills separates None from the others. It is assumed that None tends to reflect motor accuracy and dexterity and carefulness in handwriting, Mild tends to reflect clumsiness of foundational motor skills, and Severe tends to reflect clumsiness of foundational motor skills and lack of carefulness in handwriting.

### Prediction of handwriting difficulties and analysis of important indicators using LightGBM

**Fig. 4:**
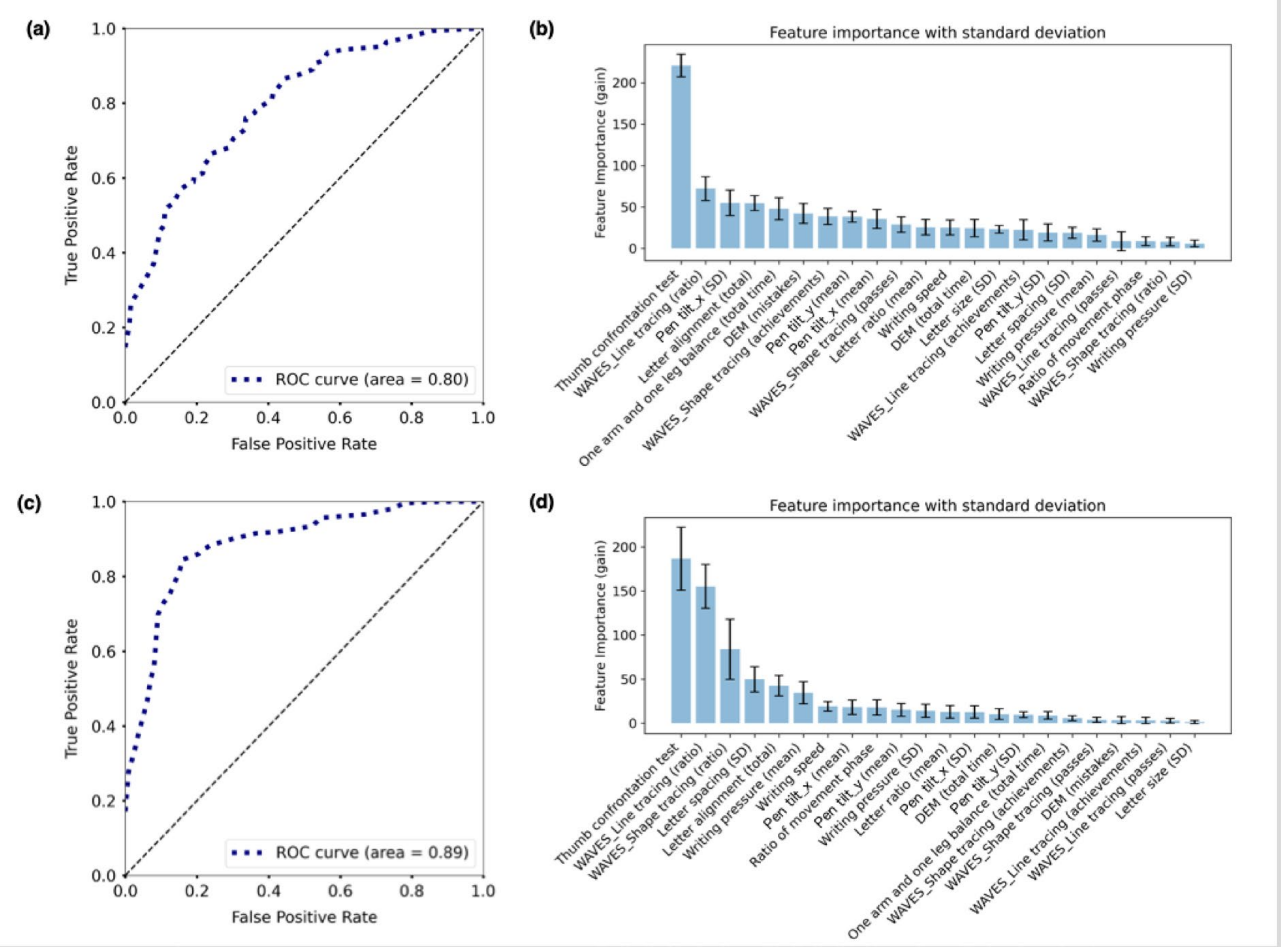
Prediction performances and distributions of feature importance obtained by the prediction models of handwriting difficulties using LightGBM. (**a**) Prediction performance for classifying None and the other groups (i.e., Mild and Severe). (**b**) Feature importance value obtained by the two-class classification of None and the other groups. (**c**) Prediction performance for classifying Severe and the other groups (i.e., None and Mild). (**d**) Feature importance values obtained by the two-class classification of Severe and the other groups.

Figure 4a shows the performance of the prediction model in classifying None and the other groups (Mild and Severe). Its accuracy and Receiver Operating Characteristic—Area Under the Curve (ROC-AUC) values are 0.72 and 0.80, respectively. The performance was evaluated using ten-fold cross-validation. Figure 4b shows the feature importance values for each assessment indicator derived from this prediction model. The most influential indicator was the thumb confrontation test, followed by WAVES_Line tracing (ratio), which relate to motor accuracy and dexterity. Among the indicators related to the assessment of the handwriting process, Pen tilt_x (SD) was important (third place). Among the indicators related to the legibility of written letters, letter alignment was highly important (fourth place).

Figure 4c depicts the performance of the prediction model in classifying Severe and the other groups (None and Mild). Its accuracy and ROC-AUC values are 0.74 and 0.89, respectively. The performance was also evaluated using tenfold cross-validation. Figure 4d shows the feature importance of each assessment indicator derived from this prediction model. The thumb confrontation test was similarly important as the results presented above. The feature importance values of WAVES_Line tracing (ratio) and WAVES_Shape tracing (ratio), which relate to lack of carefulness in handwriting, were relatively high. Among the indicators related to the legibility of written letters, letter spacing and letter alignment were important (third and fourth place).

## Discussion

The purpose of this study was to explore the relationship between handwriting difficulties based on teachers’ perceptions in educational settings and comprehensive indicators that constitute handwriting difficulties. The results of the PCA revealed that the main indicators synthesized from the 22 indicators were lack of carefulness in handwriting and clumsiness of foundational motor skills (Fig. 2). Furthermore, the relationship between teachers’ ratings of handwriting difficulties and these indicators suggested that clumsiness of foundational motor skills was associated with the presence of handwriting difficulties, whereas lack of carefulness in handwriting was associated with the severity of handwriting difficulties (Fig. 3). The results of the LightGBM-based prediction revealed that the thumb confrontation test and WAVES_Line tracing (ratio), both related to clumsiness of foundational motor skills, were important indicators for predicting handwriting difficulties. Furthermore, WAVES_Line tracing (ratio) and WAVES_Shape tracing (ratio) were important in predicting the severity of handwriting and were related to lack of carefulness (Fig. 4d). In summary, two different methods of analysis, PCA and LightGBM-based prediction, yielded similar results, suggesting that the presence of handwriting difficulties was associated with “clumsiness of foundational motor skills” and the severity of handwriting difficulties was associated with “lack of carefulness in handwriting.”

The role of coordinated movement and attention, essential components of proficient handwriting, has been reported in several studies^31,32^, and a strong association has been revealed between the lack of these abilities and handwriting difficulties^33–35^. Children with developmental coordination disorder (DCD), are reported to have poor legibility, as demonstrated by large letters, uneven spacing between letters, slow handwriting speed, and a high frequency of erasing written letters^36^. Our study also suggests that clumsiness of foundational motor skills affects the presence of handwriting difficulties among children in teachers’ evaluations, as shown in Fig. 3b and the interpretation of PC2. Children with Attention Deficit Hyperactivity Disorder (ADHD) tend to have handwriting difficulties, typically exhibiting inefficient calligraphy with fast, inaccurate, and high-pressure handwriting^37^. Our study suggests that lack of carefulness in handwriting affects not only the presence but also the severity of handwriting difficulties.

Children who are less careful in their handwriting tend to be rated Severe by teachers for the following two reasons. First, teachers tend to perceive children who lack attention, write fast, and have a messy handwriting as not attempting to improve. Compared to children rated as Mild, who may simply write clumsily and illegibly, children rated as Severe could be seen as unskilled in their approach to learning and unwilling to respond positively to instruction. As a result, children who lack carefulness in handwriting tend to be judged as Severe. The second reason relates to the difficulty of memorizing letters. Memorizing letters constitutes procedural memory, and letters are learned by repeating certain movements and storing the trajectories of those movements stored in the cerebellum^38^. However, children rated as Severe who lack carefulness in handwriting are less likely to memorize the trajectory of movements by performing fast and messy pencil manipulations, which may negatively affect letter memory.

Next, we discuss the important indicators obtained from LightGBM-based prediction models (Fig. 4). The thumb confrontation test, which assesses handwriting-related functions, was the most important indicator in the two prediction models, as shown in Fig. 4b, d. It reflects the somatosensory perception and, if there is a problem with this function, the sensations of grasping and moving the pencil become unclear, which may cause handwriting difficulties. Previous studies have also reported that handwriting problems occur when somatosensory perception is reduced^20,39^. Enhancing somatosensory feedback by, for example, placing sandpaper under the writing paper while handwriting can improve handwriting^40^. The second most important indicator in the two prediction models was WAVES_Line tracing (ratio), which reflects visuomotor integration. The relationship between handwriting ability and visuomotor integration has been repeatedly highlighted in previous studies (see the meta-analysis by Lu et al.^41^). Our findings suggest that difficulty with WAVES_Line tracing (ratio) had a greater influence than did WAVES_Shape tracing (ratio). Comparing the two WAVES tasks, it is assumed that the line tracing task, although less demanding in terms of fine finger movement, was more likely to result in fast and messy strokes and, thus, more likely to cause a discrepancy in the number of passes relative to the number of achievements. See Fig. S1 in Supplementary Information for details.

Among the indicators of legibility of written letters, letter alignment was important in predicting the presence of handwriting difficulties (classification of None and the other groups), as shown in Fig. 4b. Interestingly, the importance of letter spacing is less than that of letter alignment, but it is more important for predicting the severity of handwriting difficulties (classification of Severe and the others), as shown in Fig. 4b,d. These results suggest that the factor that distinguishes Mild from Severe is the discrepancy in letter spacing. However, letter ratio and letter size were less important in both models, despite their strong role in assessing handwriting ability, whereas letter spacing and letter alignment require processing at a higher level such as spatial cognition and executive function^42^. Our findings suggest that children who teachers perceived as having handwriting difficulties were those with difficulties that included these higher-order neural mechanisms.

Among the indicators of the handwriting process, Pen tilt_x (SD) was an important indicator of the presence of handwriting difficulties (Fig. 4b). This may indicate the instability of the upper limbs and fingers during pen manipulation. Postural stability is important for precise upper limb and finger manipulation and one arm and one leg balance, which assess postural balance function, are important indicators of the presence of handwriting difficulties.

This study has several limitations. First, it only investigated children and teachers from a single elementary school and the sample size was relatively small, limiting the generalizability of the findings. Future studies should increase the sample size and include data from multiple elementary schools. Second, the results are limited to relatively simple Japanese letters (so called hiragana and katakana). Further research is required to determine whether similar results can be obtained for other letter systems such as more complicated letters used in Japan (e.g., Chinese characters) and alphabets. Third, handwriting was performed using a digital tablet, which is different from handwriting on paper and handwriting with a pen, in terms of frictional resistance of handwriting. Future research using tools which allow paper-based assessment of handwriting is needed.

## Conclusion

This study comprehensively investigated the relationship between teachers’ perceptions of children’s difficulty in handwriting and the 22 indicators related to handwriting, categorized as the legibility of written letters, handwriting process, and handwriting-related functions. As synthesized indicators, the results suggest that clumsiness of foundational motor skills and lack of carefulness are related to the presence and severity of handwriting difficulties, respectively. As individual indicators, somatosensory and visuomotor integration are important indicators for predicting the presence of handwriting difficulties. Among the visuomotor integration indicators, the line tracing task predicted the severity of handwriting difficulties. Among the indicators related to the legibility of written letters, letter alignment and letter spacing predicted the presence and severity of handwriting difficulties, respectively. Among the indicators related to the handwriting process, Pen tilt_x (SD) was an important indicator for predicting the presence of handwriting difficulties. These results provide important guidance for teachers of children with handwriting difficulties and provide occupational therapists and other professionals a novel perspective. In the future, focusing on preschoolers as a risk factor for handwriting difficulties may enable early detection and timely support.

## Electronic supplementary material

Below is the link to the electronic supplementary material.

Supplementary Material 1

## Data Availability

The data used in this study contain personal information and are therefore not publicly available. However, the corresponding author will provide access to the data upon reasonable request.
