# Supplementary material for: Comprehensive search for assessment indicators that influence the level of handwriting difficulties among children in educational settings
Source: Sci Rep. 2025 Jul 2;15:22795. doi: 10.1038/s41598-025-03634-z (PMC12218408; doi:10.1038/s41598-025-03634-z)
Supplement: Supplementary file 1 — Supplementary Material 1 [file 41598_2025_3634_MOESM1_ESM.docx]

Supporting Information

Comprehensive search for assessment indicators that influence

the level of handwriting difficulties among children in educational settings

Shuhei Takahata^1,2*†^, Hiromichi Hagihara^3†^, Hiroyuki Ishihara^4^, Daiki Enomoto^5^, Naoto Ienaga^6^, Haruka Noda^5^, Shoichi Ishida^2^, Kei Terayama^2^*

^1^ Department of Occupational Therapy Faculty of Health Science, Aino University, 4-5-4, Higasiota, Ibaraki 567-0012, Osaka, Japan

^2^ Graduate School of Medical Life Science, Yokohama City University, 1-7-29, Suehiro-cho, Tsurumi- ku, Yokohama 230-0045, Kanagawa, Japan.

^3^ Graduate School of Human Sciences, University of Osaka, 1-2, Yamadaoka, Suita 565-0871, Osaka, Japan

^4^ Independent Researcher, Kanagawa, Japan

^5^ LITALICO Inc., 2-1-1, Kamimeguro, Meguro-ku 153-0051, Tokyo, Japan

^6^ Institute of Systems and Information Engineering, University of Tsukuba, 1-1-1, Tennoudai, Tsukuba 305-8573, Ibaraki, Japan

*Corresponding authors. E-mail(s): s-takahata@ot-u.aino.ac.jp and terayama@yokohama-cu.ac.jp

^†^These two authors contributed equally to this work.

**Assessment of handwriting-related functions used in this study**

1. Japanese Playful Assessment of Neuropsychological Abilities (JPAN)^[1]^

The JPAN was developed and standardized in Japan in 2011 as an assessment battery for sensory integration function. It assesses sensory processing and coordinated movements. It consists of four domains: posture and balance, somatosensory, praxis, and visual perception and eye-hand coordination, with a total of 32 subtests. It is characterized by its ability to comprehensively evaluate sensory processing and coordinated movements.

One of the subtests used in this study was “one arm and one leg balance test”. This test evaluates how long a participant can maintain a position in which one arm and the opposite leg are extended horizontally. It is administered twice, once for each combination: right arm with left leg, and left arm with right leg. The maximum duration for each trial is 60 seconds, resulting in a total possible score of 120 seconds across both trials.

1. Clinical Observation Assessment^[2]^

The test of “Clinical Observation” published by the Japanese Society for Sensory Integration, includes tests and observations to assess neurological soft signs. It is rated on three levels: “normal,” “slightly inferior,” and “inferior” by the therapist's clinical assessment according to the criteria of behavioral observation.

One of the assessments used in this study was the “Thumb confrontation test”. The test assesses the accuracy and smoothness of sequential tapping performed by the thumb and other fingers. Performance is evaluated based on three criteria: “normal,” “slightly inferior,” and “inferior.” In this study, scores were assigned as follows: “normal” = 3 points, “slightly inferior” = 2 points, and “inferior” = 1 point. These scores were used for analysis.

1. Developmental Eye Movement Test (DEM)^[3]^

This test assesses the accuracy and fluency of eye movements. It consists of three types of tasks: Test A, B, and C. Tests A and B involve reading aloud a sequence of 40 equally spaced numbers listed vertically (20 numbers × 2 lines), while Test C involves reading aloud a sequence of 80 randomly spaced numbers listed horizontally (5 numbers × 16 lines).

In this study, only TestC in the DEM, which imposes the highest eye movement load, was selected. Two metrics were calculated: total time, indicating eye movement fluency, and number of mistakes, indicating eye movement accuracy.

1. Wide-range Assessment of Vision-related Essential Skills (WAVES)^[4]^

This is a comprehensive assessment of visual perception and cognition. It consists of nine basic tests and one supplementary test. The scores for each test and four indices of vision-related skills (visual perception index, general eye-hand coordination index, eye-hand coordination accuracy index, and visual perception + eye-hand coordination index) are calculated. The test is characterized by its ability to assess a wide range of vision-related functions.

The two tasks used in this study, line tracing and shape tracing, are described in detail below. In the line tracing task, participants were instructed manipulate a pencil so that it remained within a designated line. See Fig.S1 for details. The line includes segments of defined width; tracing within this central segment earns 2 points, slightly outside the segment earns 1 point, and significant deviations receive 0 points. The total score is calculated as the sum of these points.

In the shape tracing task, participants were asked to trace small shapes (triangles, squares, and circles) without extending beyond their boundaries. If the tracing remained entirely within the shape, 1 point was awarded; if the tracing exceeded the shape, 0 points were given. For each task, the following three performance metrics were calculated: (1) Number of achievements – representing how far the participant progressed through the task; (2) Number of passes – indicating how accurately the pencil stayed on the intended line or shape; and (3) Pass ratio – calculated as the number of passes divided by the number of achievements.

**Reference:**

[1] The Japanese Academy of Sensory Integration. JPAN Japanese Playful Assessment for Neuropsychological Abilities Implementation Manual (Pacific Supply Inc, Osaka, 2016) (in Japanese).

[2] The Japanese academy of sensory integration. Japanese Society for Sensory Integration Disorders Certification Course Basic and Testing **8** Methods A Course Clinical Observation Manual．The Japanese academy of sensory integration.

[3] Tassinari, J. T. & DeLand, P. Developmental Eye Movement Test: reliability and symptomatology. *Optometry* **76**, 387–399 (2005).

[4] Okumura, T. *et al*. Validity of the wide-range assessment of vision-related essential skills in Japanese children with learning problems. *Optom. Vis. Sci.* **97**, 275–285 (2020).

**Figure S1.** Representative cases in WAVES_Line tracing and WAVES_Shape tracing. (a) and (b) are examples of WAVES_Line tracing, while (c) and (d) represent WAVES_Shape tracing. (a) and (b) show children achieved a high number of completions but tend to make many mistakes, resulting a low calculated ratio (number of passes/number of achievements). These children tend to write quickly and have a messy handwriting. (b) and (d) depict children achieved fewer completions but make fewer mistakes, resulting a high calculated ratio. These children tend to write slowly and carefully.
